# Supplementary material for: Sam68 splicing regulation contributes to motor unit establishment in the postnatal skeletal muscle
Source: Life Sci Alliance. 2020 Aug 4;3(10):e201900637. doi: 10.26508/lsa.201900637 (PMC7409371; doi:10.26508/lsa.201900637)
Supplement: Supplementary file 1 [file LSA-2019-00637_TableS1.docx]

**Supplementary Table 1. List of primers.**

| **Primer** | **Sequence 5’- 3’** |
| --- | --- |
| Aldh1A3_Ex9 FW | GAGTGTGGAGTTCGCCAAGA |
| Aldh1A3_Ex10 REV | GGCAATCCTCATGTTGTCCG |
| Aldh1A3_Ex6 FW | GGAACTTCCCCCTGCTGATG |
| Aldh1A3_Int7 REV | CGTCGGTGGGTCTCAAATAATGG |
| Arhgef9_FW | CAGGCTGCAATGACTGTGAGA |
| Arhgef9_REV | ACACTTGAGACTGAGCGATGC |
| CypA _FW | GTCAACCCCACCGTGTTCTT |
| CypA _REV | CTGCTGTCTTTGGGACCTTGT |
| 18S_FW | GTAACCCGTTGAACCCCATT |
| 18S_REV | CCATCCAATCGGTAGTAGCG |
| Foxo3A_FW | GCAAGCCGTGTACTGTGGA |
| Foxo3A_REV | CGGGAGCGCGATGTTATCC |
| Gphn_EX8 FW | GAGACACAGCCTCCCTTAGC |
| Gphn_EX11 REV | TGCTGCACCTGGACTGG |
| Gria2_EX11 FW | TTTCCTTGGGTGCCTTTATGC |
| Gria2_INT11 REV | ATGCACAAAGTTGAAGCAGGTG |
| Gria2_EX11EX12 FW | CAAGATCTCTCTCTGGGCGC |
| Gria2_EX12 REV | ACAGTCAGGAAGGCAGCTAA |
| Gria3 Ex12_FW | TTTCCTTGGGTGCTTTTATGCAG |
| Gria3 Ex13_REV | AGGGTGAAGAACCACCAAACC |
| Gria3 Int12_REV | TGAGGGCAGTAAGACCACAAAA |
| Lrrc7_EX22 FW | CAAACCAGGCCAGTTTCAGC |
| Lrrc7_EX27 REV | TATCCCCGGGCTGTAGTAGG |
| MAFbxAtrogin1_FW | AGCTTTGCAAACACTGCCAC |
| MAFbx/Atrogin1_REV | GAGCAGCTCTCTGGGTTGTT |
| Mhc I_FW | AGGGCGACCTCAACGAGAT |
| Mhc I_REV | CAGCAGACTCTGGAGGCTCTT |
| Mhc IIa_FW | CCAAGAAAGGTGCCAAGAAG |
| Mhc IIa_REV | CGGGAGTCTTGGTTTCATTG |
| Mhc IIb_FW | GCTTGAAAACGAGGTGGAAA |
| Mhc IIb_REV | CCTCCTCAGCCTGTCTCTTG |
| Mhc IIx_FW | CGGTGGTGGAAAGAAAGG |
| Mhc IIx_REV | CAGGAGTCTTGGTTTCATT |
| Myogenin_FW | ACTCCCTTACGTCCATCGTG |
| Myogenin_REV | ACCCAGCCTGACAGACAATC |
| MuRF1_FW | GAGGGCCATTGACTTTGGGA |
| MuRF1_REV | TTTACCCTCTGTGGTCACGC |
| Ncam2 Ex14_FW | ACCAAGCAAGATGATGGAGGG |
| Ncam2 Ex15_REV | ACCTTCTTCTCTAGCCACTGA |
| Ncam2 Ex15_FW | GTTCAGCATGCCCCCAAAAC |
| Nrxn1_FW | TGTTGGGACAGATGACATCGC C |
| Nrxn1_REV | GAGAGCTGGCCCTGGAAGGG |
| Nrxn2_FW | GTGCGCTTTACTCGAAGTGGTG |
| Nrxn2_REV | CCCATTGTAGTAGAGGCCGGAC |
| Nrxn3_FW | TTGTGCGCTTCACCAGGAATG |
| Nrxn3_REV | AGAGCCCAGAGAGTTGACCTTG |
| Sgce_FW | TATCATGTGCTGCCGACGGGAAGG |
| Sgce_REV | GTCGTAGTTGTCTGTGTGCGTGG |
| Stxbp5l_FW | CTGCAAGTCCCCCACCTCAG |
| Stxbp5l_REV | ACAAACAGACAAGGGGAAACGG |
